# Supplementary material for: Comparative Study of Pain-Related Responses of Male Piglets up to Seven Days of Age to the Application of Different Local Anaesthetics and Subsequent Castration
Source: Animals (Basel). 2022 Oct 19;12(20):2833. doi: 10.3390/ani12202833 (PMC9597853; doi:10.3390/ani12202833)
Supplement: Supplementary file 1 [file animals-12-02833-s001.zip › animals-1904905-supplementary.pdf]

animals

MDPI

Supplementary material

Comparative study of pain-related responses of male piglets up to seven days of age to the application of different local anaesthetics and subsequent castration – supplementary material

Franz Josef Söbbeler [1, *a], Sören Wendt [2, a], Andreas Briese [3], Julia Tünsmeier[1], Karl-Heinz Waldmann[2 †]; Sabine Beate Rita Kästner[1, b], Alexandra von Altrock [2, *, b]

Supplementary Data

Respiratory rate

Response to Injection

Compared to baseline measurement respiratory rate increased statistically significantly after injection in all groups. No difference was seen between the groups (Table S1).

Response to castration

During castration, the maximum respiration rate was statistically significantly higher than the basal value in all groups. After castration, the highest increase of more than 10% compared to baseline was seen in $M_{0.3}$, $S_{0.3}$ and $S_{0.5}$. Group $L_{0.5}$ was the only group without a significant increase in respiratory rate postoperatively (Table S1).

Mean arterial blood pressure

Response to Injection

Upon injection mean arterial blood pressure increased in all groups. Compared to intramuscular injection the change of MAP was statistically significant only in group $P_{0.3}$ and $S_{0.5}$ (Table S1).

Response to castration

Group $L_{0.5}$ and group $M_{0.5}$ were the only groups without significant increase in MAP during castration compared to baseline castration. In $P_{0.5}$ only a small increase was detected during emasculation. In all other groups the blood pressure started to increase significantly during emasculation and post operatively with the biggest increases in group $M_{0.3}$ and both placebo groups (Table S1).

Table S1. Mean and standard deviation of respiratory rate [breaths min^{-1}], mean arterial blood pressure (MAP [mmHg]), and pulse rate [pulses min^{-1}]) for groups $P_{0.3}$, $P_{0.5}$, $L_{0.3}$, $L_{0.5}$, $M_{0.3}$, $M_{0.5}$, $S_{0.3}$, $S_{0.5}$ and IM for the timepoints baseline Injection (BL-INJ), injection (INJ), baseline castration (BL-CA), castration (CA) and post castration (POST-CA). # represents a significant difference to baseline ($p < 0.05$).

parameter		group	BL-INJ	INJ-MAX	BL-CA	skin incision	exteriorization of testicles	emasculation	CA-MAX	POST-CA
respiratory rate [breaths min-1]	$\bar{x} \pm s$	**$P_{0.3}$**	36,4 ± 9,9	39,9 ± 8,7 #	37,8 ± 10,7	38,1 ± 10,2	38,7 ± 10,0	40,4 ± 10,2 #	41,3 ± 10,1 #	41,4 ± 10,7 #
		**$P_{0.5}$**	32,6 ± 8,3	37,8 ± 7,2 #	32,4 ± 6,6	32,5 ± 6,8	33,0 ± 6,4 #	34,2 ± 6,0 #	34,9 ± 6,1 #	33,2 ± 6,0 #
		**$L_{0.3}$**	32,1 ± 7,8	35,4 ± 8,2 #	31,6 ± 8,0	31,8 ± 8,2	32,6 ± 8,6 #	33,6 ± 8,4 #	34,4 ± 8,3 #	32,8 ± 8,0 #
		**$L_{0.5}$**	30,7 ± 7,2	36,5 ± 7,5 #	30,1 ± 6,3	30,4 ± 6,5 #	30,7 ± 6,4 #	31,5 ± 6,7 #	31,8 ± 6,9 #	30,5 ± 6,2
		**$M_{0.3}$**	35,1 ± 11,3	39,2 ± 10,6 #	36,3 ± 12,0	36,6 ± 11,9	37,9 ± 11,9 #	39,6 ± 12,3 #	40,6 ± 12,0 #	42,3 ± 11,4 #
		**$M_{0.5}$**	32,5 ± 6,2	36,5 ± 6,0 #	33,0 ± 6,4	33,2 ± 6,5	33,6 ± 6,6 #	34,2 ± 6,7 #	35,0 ± 6,6 #	34,4 ± 6,6 #

		**$S_{0.3}$**	32,6 ± 5,9	36,3 ± 6,1 #	34,0 ± 5,9	34,6 ± 5,7 #	35,7 ± 6,6 #	37,1 ± 6,9 #	38,2 ± 6,8 #	39,9 ± 6,5 #
		**$S_{0.5}$**	33,9 ± 7,1	39,5 ± 5,4 #	35,1 ± 7,5	35,6 ± 7,3 #	37,8 ± 8,5 #	39,0 ± 7,7 #	39,8 ± 7,7 #	40,8 ± 6,3 #
		**IM**	32,5 ± 5,0	34,9 ± 4,6 #						
MAP [mmHg]	$\bar{x} \pm s$	**$P_{0.3}$**	47,0 ± 5,2	54,9 ± 6,2 #	50,3 ± 5,5	50,9 ± 5,6 #	50,9 ± 5,1	54,6 ± 6,6 #	61,6 ± 8,5 #	59,5 ± 8,1 #
		**$P_{0.5}$**	47,0 ± 4,6	52,4 ± 6,3 #	45,9 ± 4,9	45,7 ± 4,7	45,4 ± 4,6	48,8 ± 5,8 #	50,0 ± 6,4 #	47,4 ± 5,2
		**$L_{0.3}$**	46,9 ± 5,6	51,2 ± 6,0 #	46,8 ± 5,6	46,8 ± 5,7	46,6 ± 5,5	49,1 ± 6,9 #	50,9 ± 6,0 #	49,4 ± 5,8 #
		**$L_{0.5}$**	48,3 ± 5,1	53,9 ± 6,1 #	47,1 ± 5,4	46,4 ± 4,9	46,6 ± 5,4	48,0 ± 5,6	48,9 ± 6,2 #	47,2 ± 6,3
		**$M_{0.3}$**	49,8 ± 6,1	52,2 ± 5,3 #	50,0 ± 6,6	50,3 ± 6,4	49,5 ± 5,9	54,1 ± 6,0 #	60,0 ± 7,8 #	68,8 ± 10,9 #
		**$M_{0.5}$**	50,6 ± 6,6	56,8 ± 7,2 #	51,6 ± 5,5	51,9 ± 5,1	51,3 ± 5,3	53,3 ± 5,6	54,8 ± 5,2 #	50,8 ± 5,3
		**$S_{0.3}$**	50,1 ± 8,2	56,5 ± 8,7 #	50,8 ± 5,9	50,7 ± 5,8	51,5 ± 5,9	58,5 ± 10,1 #	64,0 ± 10,7 #	71,6 ± 9,5 #
		**$S_{0.5}$**	47,9 ± 9,7	56,3 ± 10,1 #	49,3 ± 10,4	49,6 ± 10,1	51,1 ± 10,5	56,2 ± 13,1 #	61,6 ± 12,5 #	68,6 ± 13,7 #
		**IM**	45,4 ± 7,7	46,7 ± 8,6 #						
Pulserate [Pulses min-1]	$\bar{x} \pm s$	**$P_{0.3}$**	171,0 ± 24,0	180,2 ± 25,6 #	170,2 ± 24,4	170,0 ± 24,3	172,1 ± 25,2 #	178,6 ± 26,3 #	184,2 ± 28,8 #	176,3 ± 24,8 #
		**$P_{0.5}$**	169,6 ± 20,5	184,5 ± 26,0 #	160,5 ± 19,8	160,4 ± 20,1	160,7 ± 19,4	163,8 ± 19,6 #	166,9 ± 21,5 #	164,3 ± 20,1 #
		**$L_{0.3}$**	176,4 ± 19,5	182,6 ± 22,9 #	174,4 ± 21,3	173,9 ± 20,8	173,7 ± 21,5	177,5 ± 23,9 #	179,8 ± 23,4 #	175,7 ± 21,7
		**$L_{0.5}$**	180,5 ± 21,4	194,4 ± 25,3 #	166,6 ± 18,7	166,7 ± 19,3	166,9 ± 19,6	167,1 ± 19,4	168,6 ± 19,7 #	167,0 ± 19,5
		**$M_{0.3}$**	172,9 ± 21,1	178,2 ± 21,7 #	173,9 ± 22,2	174,1 ± 22,0	173,9 ± 21,4	184,2 ± 24,1 #	190,6 ± 23,9 #	177,0 ± 22,0 #
		**$M_{0.5}$**	182,1 ± 25,6	194,8 ± 29,4 #	173,4 ± 24,6	173,8 ± 25,2	173,9 ± 24,8	175,5 ± 23,8	176,7 ± 25,0 #	173,6 ± 25,7
		**$S_{0.3}$**	180,4 ± 22,3	186,7 ± 22,9 #	177,7 ± 20,2	178,5 ± 20,1	180,7 ± 20,9 #	191,6 ± 24,3 #	198,4 ± 23,3 #	182,0 ± 21,2 #
		**$S_{0.5}$**	169,5 ± 25,6	180,1 ± 27,2 #	168,9 ± 29,6	169,8 ± 28,9	171,2 ± 28,2	180,6 ± 32,0 #	188,5 ± 31,9 #	176,4 ± 28,7 #
		**IM**	181,6 ± 26,1	184,9 ± 28,3 #						

Heart rate variability

Heart rate

Response to injection

Compared to baseline measurement a statistically significantly increase in heartrate upon injection was seen in groups $M_{0.5}$ and $S_{0.5}$ (Table S2).

Response to castration

A statistically significantly increase in heartrate during castration was seen in all groups except $L_{0.5}$ and $M_{0.5}$ wheras after castration heartrate of both procaine and both saline groups was still statistically significantly above baseline. In group $L_{0.5}$ heartrate decreases statistically significantly after castration (Table S2).

Mean RR-Interval

Response to injection

Compared to baseline measurement a statistically significant decrease in mean RR-intervals upon injection was seen in groups $L_{0.5}$ and $S_{0.5}$ (Table S2).

Response to castration

A statistically significant decrease in RR-intervals was noted during castration in all groups but $L_{0.5}$ and $M_{0.5}$. Postoperatively both procaine and both saline groups showed a decrease in RR-intervals. A statistically significant increase in mean RR-intervals was seen after castration in group $L_{0.5}$ (Table S2).

SDHR and SDRR

Response to injection

The SDHR and the SDRR increased in all groups upon injection except $L_{0.5}$ and IM (Table S2).

Response to castration

During castration the SDHR and the SD RR increased in all groups but $M_{0.5}$ and after castration no statistically significant differences were seen compared to baseline in any group (Table S2).

LF/HF-ratio

Response to injection

The LF/HF-ratio increased in all groups upon injection. The mildest increase (to 138% compared to baseline) was seen during intramuscular injection whereas the highest increase was seen during injection in group $P_{0.5}$ (1328%) (Table S2).

Response to castration

During castration a statistically significant increase was seen in all groups but $M_{0.5}$. No significant differences were seen during the postoperative measurement period in any group (Table S2).

Table S2. Heart rate variability. Mean and standard deviation of heart rate [beat min^{-1}], RR-Interval [ms], and median, minimum and maximum of Standard deviation of heartrate (SD HR [beat min^{-1}]), Standard deviation of RR-Interval (SD RR [ms]) and Low Frequency High Frequency ratio (LF/HF-ratio) for groups $P_{0.3}$, $P_{0.5}$, $L_{0.3}$, $L_{0.5}$, $M_{0.3}$, $M_{0.5}$, $S_{0.3}$, $S_{0.5}$ and IM for the timeperiods baseline Injection (BL-INJ^e), injection (INJ^e), baseline castration (BL-CA^e), castration (CA^e) and post castration (POST-CA^e). # represents a significant difference to baseline ($p < 0.05$).

parameter		group	BL-INJ^e	INJ^e	BL-CA^e	CA^e	POST-CA^e
heart rate [beats min-1]	$\bar{x} \pm s$	$P_{0.3}$	179,1 ± 27,1	179,6 ± 26,7	177,0 ± 27,4	182,4 ± 27,8 #	183,9 ± 28,6 #
		$P_{0.5}$	170,3 ± 25,7	172,5 ± 26,7	159,4 ± 23,1	162,2 ± 24,0 #	163,5 ± 24,1 #
		$L_{0.3}$	177,4 ± 27,5	176,9 ± 26,0	174,6 ± 27,7	176,2 ± 28,1 #	175, ± 26,61
		$L_{0.5}$	179,8 ± 20,6	182,4 ± 21,5	167,6 ± 18,6	166,8 ± 18,9	165,9 ± 19,1 #
		$M_{0.3}$	177,2 ± 21,0	176,3 ± 19,7	176,4 ± 22,2	182,8 ± 21,4 #	178,8 ± 20,5
		$M_{0.5}$	180,6 ± 25,1	182,8 ± 25,8 #	173,8 ± 24,4	174,5 ± 24,0	173,6 ± 24,8
		$S_{0.3}$	180,9 ± 20,1	179,8 ± 17,6	178,7 ± 19,9	186,6 ± 20,9 #	182,2 ± 21,0 #
		$S_{0.5}$	170,5 ± 24,3	173,4 ± 24,1 #	170,6 ± 26,4	178,4 ± 28,3 #	176,6 ± 26,4 #
		IM	180,5 ± 23,8	180,5 ± 24,4			
RR-interval [ms]	$\bar{x} \pm s$	$P_{0.3}$	342,52 ± 53,17	341,35 ± 52,35	346,91 ± 54,03	336,55 ± 52,59 #	334,00 ± 52,37 #
		$P_{0.5}$	362,47 ± 73,31	358,16 ± 72,02	386,02 ± 71,98	380,02 ± 74,26 #	376,99 ± 73,81 #
		$L_{0.3}$	346,03 ± 53,28	346,32 ± 52,35	351,95 ± 55,78	348,80 ± 55,39 #	349,21 ± 51,63
		$L_{0.5}$	338,26 ± 43,02	333,61 ± 42,57 #	362,64 ± 44,07	364,50 ± 45,19	366,71 ± 46,60 #
		$M_{0.3}$	343,39 ± 42,78	344,64 ± 40,47	345,61 ± 46,02	332,81 ± 41,45 #	340,12 ± 41,41
		$M_{0.5}$	338,99 ± 51,49	334,90 ± 50,75	352,43 ± 54,20	350,90 ± 53,91	353,20 ± 55,92
		$S_{0.3}$	335,15 ± 33,24	336,63 ± 30,71	339,40 ± 34,50	325,18 ± 34,83	333,26 ± 36,07 #
		$S_{0.5}$	359,95 ± 60,19	353,73 ± 59,73 #	361,90 ± 71,95	346,57 ± 70,31 #	349,19 ± 68,41 #
		IM	338,34 ± 47,87	338,58 ± 48,96			
SD HR [beats min-1]	$\tilde{x}$ (Min; Max)	$P_{0.3}$	0,65 (0,47; 1,65)	1,06 (0,71; 2,17) #	0,7 (0,46; 1,73)	1,42 (0,84; 2,94) #	0,74 (0,47; 1,49)
		$P_{0.5}$	0,61 (0,33; 1,25)	1,16 (0,40; 2,46) #	0,62 (0,26; 1,11)	1,07 (0,26; 2,64) #	0,66 (0,22; 0,99)
		$L_{0.3}$	0,71 (0,48; 1,06)	0,91 (0,55; 2,56) #	0,7 (0,46; 1,63)	1,3 (0,51; 1,97) #	0,77 (0,42; 1,39)
		$L_{0.5}$	0,83 (0,07; 2,48)	1,05 (0,67; 2,63)	0,62 (0,37; 1,35)	0,86 (0,42; 2,43) #	0,65 (0,40; 1,02
		$M_{0.3}$	0,77 (0,45; 1,54)	1,03 (0,51; 1,82) #	0,86 (0,45; 1,31)	1,87 (1,21; 5,56) #	0,85 (0,52; 1,28)
		$M_{0.5}$	0,74 (0,41; 1,32)	0,94 (0,56; 1,77) #	0,78 (0,47; 1,35)	0,89 (0,47; 2,46)	0,77 (0,44; 1,84)
		$S_{0.3}$	0,76 (0,06; 1,05)	1,02 (0,56; 2,31) #	0,77 (0,48; 1,09)	2,07 (0,83; 6,87) #	0,87(0,48; 1,60)
		$S_{0.5}$	0,63 (0,35; 1,62)	0,97 (0,41; 2,18) #	0,73 (0,37; 1,24)	1,91 (0,74; 3,42) #	0,81 (0,43; 1,61)
		IM	0,63 (0,40; 3,04)	0,74 (0,37; 1,37)			
SDNN [ms]	$\tilde{x}$ (Min; Max)	$P_{0.3}$	1,51 (0,87; 3,01)	2,12 (1,34; 3,24) #	1,53 (0,90; 2,38)	3,07 (1,55; 4,32) #	1,3 (0,80; 3,02)
		$P_{0.5}$	1,24 (0,90; 2,11)	2,3 (1,19; 4,73) #	1,51 (0,95; 4,42)	2,26 (0,96; 5,12) #	1,5 (0,98; 2,44)
		$L_{0.3}$	1,33 (0,86; 3,22)	1,81 (0,77; 3,68) #	1,49 (0,75; 3,00)	2,41 (0,96; 4,67) #	1,43 (0,76; 3,66)
		$L_{0.5}$	1,75 (1,06; 5,53)	2,05 (1,10; 4,06)	1,35 (0,86; 2,67)	1,71 (0,96; 3,85) #	1,51 (0,86; 2,29)
		$M_{0.3}$	1,54 (0,79; 2,39)	1,81 (0,89; 3,42) #	1,56 (0,90; 2,72)	4,03 (1,85; 8,76) #	1,53 (1,03; 2,49)
		$M_{0.5}$	1,15 (0,85; 2,55)	1,66 (1,12; 2,77) #	1,55 (0,95; 2,74)	1,68 (0,96; 5,67)	1,52 (0,92; 3,57)
		$S_{0.3}$	1,38 (0,83; 2,14)	1,9 (1,12; 4,60) #	1,46 (1,06; 2,06)	3,26 (1,66; 12,10) #	1,6 (0,91; 2,37)

		**S0.5**	1,31 (0,87; 2,93)	2,05 (1,00; 4,73) #	1,35 (0,90; 7,68)	3,25 (1,23; 11,36) #	1,6 (0,90; 6,70)
		**IM**	1,22 (0,93; 3,50)	1,39 (0,87; 2,10)			
LF/HF-ratio	~x (Min; Max)	**P0.3**	0,11 (0,01; 7,37)	0,96 (0,05; 4,69) #	0,22 (0,01; 10,32)	2,42 (0,53; 8,83) #	0,21 (0,04; 1,85)
		**P0.5**	0,07 (0,01; 0,49)	0,93 (0,07; 7,83) #	0,11 (0,00; 1,33)	0,66 (0,01; 16,71) #	0,17 (0,02; 0,77)
		**L0.3**	0,1 (0,01; 1,62)	0,56 (0,05; 2,25) #	0,1 (0,02; 0,90)	0,96 (0,04; 7,79) #	0,14 (0,03; 4,53)
		**L0.5**	0,1 (0,02; 3,42)	0,62 (0,14; 13,07) #	0,06 (0,02; 1,15)	0,09 (0,03; 23,44) #	0,08 (0,01; 0,69)
		**M0.3**	0,1 (0,01; 1,56)	0,56 (0,08; 2,43) #	0,16 (0,02; 1,63)	4,36 (0,63; 18,59) #	0,29 (0,04; 1,76)
		**M0.5**	0,07 (0,01; 1,22)	0,32 (0,06; 2,08) #	0,07 (0,02; 3,06)	0,22 (0,01; 24,97)	0,09 (0,01; 13,99)
		**S0.3**	0,13 (0,01; 0,65)	0,85 (0,03; 9,52) #	0,24 (0,01; 1,52)	5,28 (0,04; 45,42) #	0,48 (0,07; 2,87)
		**S0.5**	0,08 (0,00; 1,37)	1,05 (0,09; 6,49) #	0,15 (0,04; 1,05)	3,47 (0,36; 26,48) #	0,22 (0,09; 1,41)
		**IM**	0,13 (0,02; 0,60)	0,18 (0,04; 2,18) #			
